# 

**Published:** 1806-03

**Authors:** 


### TO CORRESPONDENTS.

Mr. Trye's communication on the comparative Merits of Cow-pock and Small-pox Inoculation, arrived too late for insertion in this Number, but will certainly appear in our next. An Analysis of Mr. Fox's Work on the Diseases of the Teeth will also be given in our next Journal.

Communications are likewise received from Mr. Weaver, Mr. Marson, and Mr. Fogo.

